# An Observational Study Examining the Relationship between Respiratory Symptoms, Airway Inflammation and Bacteriology in Children with Severe Neurodisability

**DOI:** 10.1371/journal.pone.0124627

**Published:** 2015-04-08

**Authors:** Ruth E. Trinick, Lara Bunni, Kent Thorburn, Angela P. Hackett, Mark Dalzell, Paul S. McNamara

**Affiliations:** 1 Alder Hey Children’s Hospital NHS Foundation Trust, Eaton Rd, Liverpool, United Kingdom; 2 Institute of Translational Medicine (Child Health), University of Liverpool, Alder Hey Children’s Hospital, Eaton Road, Liverpool, United Kingdom; University Hospital San Giovanni Battista di Torino, ITALY

## Abstract

**Background:**

Children with severe neurodisability (ND) commonly suffer from chronic respiratory symptoms that impact greatly on quality of life, and lead to recurrent hospital admissions. This morbidity (and its causes) is poorly described, despite being well recognised by paediatricians. In this study, we characterised respiratory symptoms in children with ND at times of stability and deterioration. We also assessed the relationship between respiratory symptoms, lower airway inflammatory markers and levels of infection/colonisation.

**Methods:**

ND children were recruited upon admission for elective surgery (Elective-ND [n = 16]), or acutely upon admission to Intensive Care (PICU-ND [n = 19]), and compared to healthy control children [n = 12]. Parents completed a validated respiratory symptom questionnaire in which symptoms associated with activity were removed (total maximal score of 108). Bronchoalveolar lavage (BAL) was collected, and BAL neutrophil counts, IL-8 and TGFβ-1 levels measured. BAL microbial analysis was performed using a 16S/18S rRNA gene based assay and *Pseudomonas aeruginosa* PCR.

**Results:**

All ND children had high levels of respiratory symptoms (median [IQR] symptom score PICU-ND, 55[38-64]; Elective-ND, 26[7-45]; Control, 4[0-7]: p<0.01), which affected their families, particularly at nighttime. Elective-ND patients with a total respiratory symptom score >20 invariably had BAL neutrophilia. Elective patients with 16S/18S microbial rDNA positive BAL had higher neutrophil counts (positive, 33[18-70]%; negative, 8[4-38]%: p<0.05) and generally higher symptom scores (positive, 17[5-32]; negative, 5[0-9]: p = 0.097). *Streptococcus mitis* was commonly identified in BAL from ND children; *Pseudomonas aeruginosa* was not identified in any sample.

**Conclusions:**

Children with severe ND often have high levels of chronic respiratory symptoms, which may relate to lower airway inflammation. Bacterial airway colonisation, particularly with oral commensals, may play a role in both symptom generation and inflammation.

## Introduction

Children with severe neurodisability (ND) often have significant respiratory morbidity.[1] Although this is well recognised by paediatricians, the respiratory burden of disease in this group of patients has never been formally documented and described. It is known however, that children with ND are the second commonest paediatric users of home oxygen after children with chronic neonatal lung disease, and respiratory complications are the leading cause of premature death.[2,3] Children with severe neurological impairment with chronic pulmonary aspiration have a high prevalence of bronchiectasis and fibrosis on chest computerised tomography scans.[4] Commonly, recurrent episodes of deterioration, often caused or complicated by infection, lead to repeated hospital admissions, impacting greatly on patients, families and health services.

There is limited information about lower airway microbiology or levels of inflammation in children with ND, either during respiratory exacerbations or when otherwise well. Antibiotic treatment during acute episodes is often prescribed empirically, without evidence of the causative organism. Despite this, antibiotic prophylaxis is becoming an accepted part of the clinical management of children with ND with recurrent respiratory symptoms and difficulties.

As part of a larger study on aspiration lung disease, we documented the burden of respiratory symptoms in children with severe neurodisability using a respiratory symptom score, at a time of both clinical stability and respiratory deterioration. To investigate the relationship between these symptoms and lower airway inflammatory levels, we analysed Interleukin-8 (IL-8, a potent neutrophil chemoattractant) and Transforming Growth Factor-β1 (TGFβ-1, a protein important in wound repair, airway remodelling and the development of sub-epithelial fibrosis). We also analysed the relationship between symptoms and airway infection by retrospectively using 16S microbiome analysis to identify predominant organisms within the lower airways.

## Methods

### Patients

This study was undertaken at Alder Hey Children’s Hospital in Liverpool, UK between October 2009 and September 2011. Prior to the start of the study, the Liverpool Paediatric Research Ethics Committee reviewed and approved the study and consent procedure on the 23rd July 2009 (REC Reference number: 09/H1002/58). As only children <16 years of age were recruited, informed written parental consent and patient assent (when appropriate) were obtained. Children over the age of two years with central ND (not neuromuscular disease) who were non-ambulant (Gross Motor Function Classification IV-V), were recruited either at a time of respiratory stability when admitted for elective surgical procedures, or at a time of respiratory deterioration when admitted and ventilated on the paediatric intensive care unit (PICU). Demographic details, previous respiratory management, clinical sample microbiology results and medication history were recorded.

As a comparative group, healthy control children over the age of two were also recruited when admitted for routine elective minor surgical procedures. Children were not recruited if they had any chronic illness or were taking any regular medications.

### Respiratory Symptom Questionnaire

Parents of all participants provided information about their child’s recent respiratory symptoms by completing the Liverpool Respiratory Symptom Questionnaire (LRSQ), previously validated in preschool children with wheeze, and children with cystic fibrosis.[5,6] The questionnaire covers respiratory symptoms over the previous three months and is divided into eight domains; daytime, night-time, interval, activity and other respiratory symptoms, symptoms with colds, the effect of symptoms on the child, and the effect of symptoms on the family. As by definition, the ND study group were not independently mobile, we removed the ‘activity symptoms’ domain and a question regarding physical activity in the ‘effect on the child’ domain for the purposes of this study, thus creating a modified questionnaire (LRSQ-Neuro). Potential scores for the LRSQ-Neuro range from 0–108.

### Sample collection and processing

Non-bronchoscopic bronchoalveolar lavage samples were collected according to European Respiratory Society (ERS) guidelines,[7] following induction for elective surgery in the anaesthetic room, or within 48 hours of the child being muscle relaxed and sedated on PICU. Briefly, a suction catheter was passed down the endotracheal tube until resistance was felt. Two 1ml/kg aliquots of normal saline were instilled separately. BAL fluid was recovered with constant suction pressure and collected into a mucus trap. The sample was then centrifuged at 2656xg, and the supernatant removed and stored at -80°C.

### Differential Cell and Cytokine Analysis

Cytospin slides were prepared from whole BAL. Following fixation in Diff Quick fixative (Reastain CE), they were stained with Diff Quick stain according to the manufacturer’s instructions. A minimum of 300 cells was counted on three occasions and a mean BAL differential cell count recorded.

Interleukin 8 (IL-8) and Transforming Growth Factor Beta 1 (TGFβ-1) were measured in BAL supernatants using commercially available ELISA kits (R&D Systems UK).

### Bacterial/Fungal DNA Isolation and 16S/18S rDNA PCR Analysis and Sequencing

Routine culture of BAL samples was not performed on BAL samples at the time of collection and so microbiological analysis was done retrospectively using 16S/18S PCR analysis and sequencing. BAL samples were analysed in the Molzym laboratories in Germany using the UMD-Liquid test (Molzym, Bremen), a variant of the SepsiTest kit designed for extraction of DNA and PCR analysis of body liquids other than whole blood. This technique tests for 345 organisms (full list available at http://www.sepsitest.com/lab-diagnostics.html) and includes a sample pre-treatment step in which human and/or microbial extracellular DNA is degraded, leaving only DNA from microbes with intact cell walls to be extracted and purified.[8] This test identifies only predominant organisms within a sample, and has a lower detection limit of ≤ 10 genome equivalents per PCR reaction. The PCR analysis comprises a universal rRNA gene-based assay for bacteria (16S) and fungi (18S). For positive results, amplicons are purified and sequences analysed.

In our study, we analysed BAL stored in microfuge tubes rather than the recommended Molzym UMD tubes. To ensure that this did not adversely affect results, we prospectively analysed paired BAL samples stored in microfuge tubes and UMD tubes for six patients. We detected the same microbes in both tubes, but storing samples in microfuge tubes appeared to decrease assay sensitivity, with microbe detection occurring a mean of four PCR cycles later (data not shown).

### Bacterial DNA Extraction and OprL Gene Analysis

To corroborate the results of the 16S rDNA assay and to check for the presence of *Pseudomonas aeruginosa* DNA, a semi-quantitative PCR was undertaken using a previously validated, specific PCR protocol based on an outer membrane lipoprotein gene (OprL).[9] Bacterial DNA was extracted from 0.2mls of BAL using the DNeasy Blood and Tissue Kit following the Gram Negative Bacterial Protocol (Qiagen, UK). The primer sequences were PAL1 (forward), 5’-ATGGAAATGCTGAAATTCGGC and PAL2 (reverse), 5’-CTTCTTCAGCTCGACGCGACG. The PCR mix consisted of 5ul of GoTaq Flexi Buffer (Promega), 0.5ul 10mM dNTPs (Bioline), 0.75ul PAL1, 0.75ul PAL2, 2.5ul MgCl_2_ (Promega), 0.25ul GoTaq Flexi DNA polymerase (Promega), 14.25ul H_2_0 and 1ul DNA per reaction. All experiments included a positive control with *P*. *aeruginosa* DNA taken from bacterial colonies, a known *P*. *aeruginosa* culture positive cystic fibrosis BAL DNA sample (processed by identical methods to the study samples) and a negative control without DNA. Analysis was performed on BAL samples that had been centrifuged for 5 minutes at 2656g.

PCR on BAL for eukaryotic 18S ribosomal RNA (from either host inflammatory cells or airway epithelial cells) was also performed to confirm that we were extracting DNA adequately from our samples.

### Statistical Analysis

SPSS version 19.0 was used. Data are expressed as median and inter-quartile range [IQR]. The Mann Whitney U test was used for comparison of ages between groups. The Chi Squared test was used for comparison of gender between groups. The Mann Whitney U test was used to look for statistical differences in continuous data between groups. Spearman’s rank correlation was used to examine the correlation of LRSQ-Neuro scores with BAL inflammatory levels.

## Results

### Clinical Characteristics

Nineteen children with ND were recruited when ventilated for respiratory failure on PICU (PICU-ND), and 16 different children with ND were recruited when otherwise well, on admission for routine elective surgery (Elective-ND). Twelve healthy control children were also recruited. There were no significant differences in median age or gender between the groups (Table 1).

**Table 1:** Basic demographic, and broncho-alveolar lavage cellular/cytokine characteristics.

	Healthy Controls(N = 12)	Elective ND(N = 16)	PICU ND (N = 19)
Age (years)	7.5 [5–11.75]	10 [2.25–14.5]	7 [5–11]
Gender (% male)	50	50	46
Total cell concentration (x 10^6^ cells/ml)	3.9[1.3–7.4]	0.9[0.4–4.2]	0.6[0.3–0.9]
Neutrophils (%)	6.8[3–18.5]	48.8[29.1–71.2]	82[72–86.6]
Alveolar macrophages (%)	89.5[78.2–94.3]	47.5[24–69]	15.7[10.4–19.8]
Lymphocytes (%)	1.8[1.4–2.7]	2.0[1.3–3]	1.7[1.3–3.2]
Eosinophils (%)	0.3[0.1–0.6]	0.3[0–0.6]	0.3[0–0.6]
Total protein (mcg/ml)	73[49–160]	124[83–694]	579[180–1412]
IL-8 (pg/ml)	0 [0–222]	646 [0–3633]	5,641 [2,024–14,703]
TGF-1 (pg/ml)	0 [0–0]	0 [0–143]	157 [69–190]

Median (IQR) cellular, protein and cytokine components of BAL are shown.

All children with ND were in Gross Motor Function Classification Groups IV/V and none had tracheostomies. In the PICU-ND and Elective-ND groups, 44% and 56% of children had cerebral palsy, respectively, with the remaining children having other central neurological diagnoses. 24/35 (68.5%) ND patients had been treated surgically or medically for gastro-oesophageal reflux disease, 16/35 (45.7%) had evidence of aspiration on videofluoroscopy, and 21/35 (60%) patients were non-orally fed. For those patients admitted to PICU with an acute respiratory exacerbation, the median [range] time to BAL sample collection following endotracheal intubation was 15 [12–22] hours.

Table 2 shows some of the microbiological and clinical characteristics of the Elective-ND and PICU-ND patients. Briefly, most PICU-ND patients had previous and/or current admission airway microbiology results. 12/16 had cultured *P*. *aeruginosa* (some on multiple occasions) on sputum or tracheal aspirate samples. 5/16 cultured *P*. *aeruginosa* on tracheal aspirate on admission to PICU. Few patients were receiving anti-pseudomonal treatment. Most ND patients had never received a specialist respiratory paediatric opinion. Many of those being cared for by respiratory specialists were on home non-invasive ventilation or home oxygen. 10/19 PICU-ND patients were prescribed prophylactic antibiotics; treatment varied and included azithromycin (n = 4), amoxicillin (n = 2), co-amoxiclav (n = 1), trimethoprim (n = 2), cefaclor (n = 1). Most PICU-ND patients had been on treatment doses of antibiotics for more than 24 hours prior to admission.

**Table 2:** Microbiological and clinical characteristics of Elective ND and PICU ND patients.

	Elective ND (N = 16)	PICU ND (N = 19)
**Cerebral Palsy (%)**	56	42
Number of patients in whom microbiological testing of respiratory samples had been undertaken (Tracheal Aspirate [TA], Sputum [S])	1/16 (1 TA)	16/19 (13 TA, 3 S)
Number of patients in whom *P*. *aeruginosa* had been detected in respiratory samples	1/1	12/16
Number of patients in whom *P*. *aeruginosa* had been detected during the current admission	N/A	5/16
**Number of patients receiving:**		
Specialist respiratory paediatric care	1/16	7/19
Home oxygen	0/16	7/19
Home non-invasive ventilation	0/16	3/19
Prophylactic antibiotics	2/16	10/19
Anti-Pseudomonal therapy	1/16	2/19
Antibiotic therapy in 24 hours prior to recruitment	0/16	13/19

(No patients had tracheostomies in place)

Only 1/16 Elective-ND patient had previous airway microbiology results. This patient had previous *P*. *aeruginosa* detected on multiple sputum and tracheal aspirate samples. 2/16 Elective-ND patients were prescribed antibiotic prophylaxis (cotrimoxazole (n = 1), trimethoprim (n = 1)).

### LRSQ-Neuro Scores

**Fig 1:**
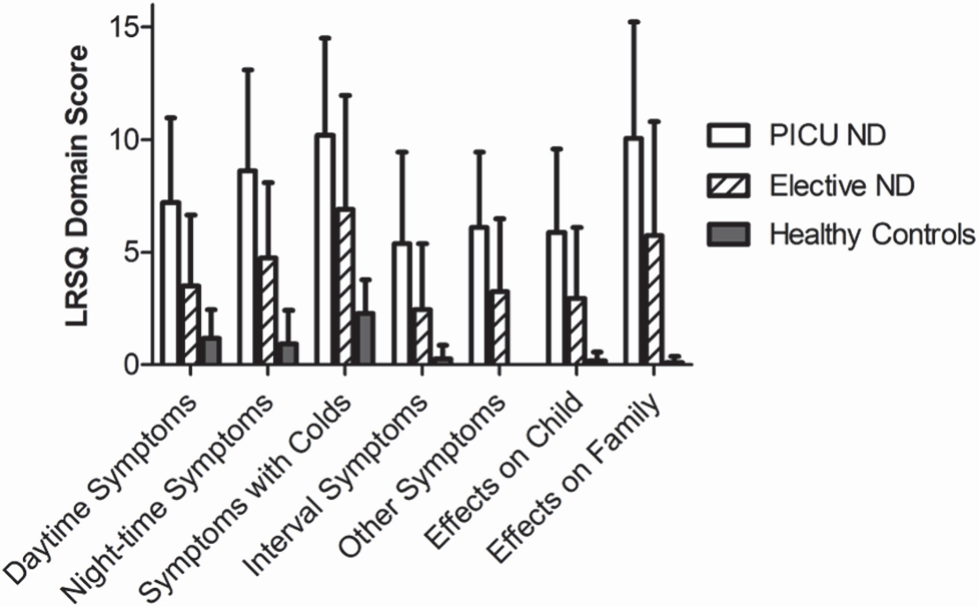
Mean (SEM) LRSQ-Neuro domain scores in PICU-ND, Elective-ND and healthy control patients over previous three months. Elective-ND patients scored significantly higher than healthy controls in all LRSQ-Neuro domains, except ‘Symptoms with colds’ (p<0.05).

As anticipated, the PICU-ND group reported most respiratory symptoms over the three months prior to admission to hospital, with median [IQR] LRSQ-Neuro scores of 55[38–64]. However, high levels of respiratory symptoms were also reported in the Elective-ND group compared to healthy controls (ND elective, 26[7–45]; Control, 4[0–7]: p<0.01). Individual domain scores in Elective-ND patients were significantly higher than healthy controls across all domains, except ‘Symptoms with colds’. ‘Effect of symptoms on the family’ (p = 0.001), ‘Other symptoms’ (p = 0.001) and ‘Nighttime symptoms’ (p = 0.004) reached the highest levels of statistical significance (Fig 1).

### Broncho-alveolar lavage cellularity and differential cell counts

**Fig 2:**
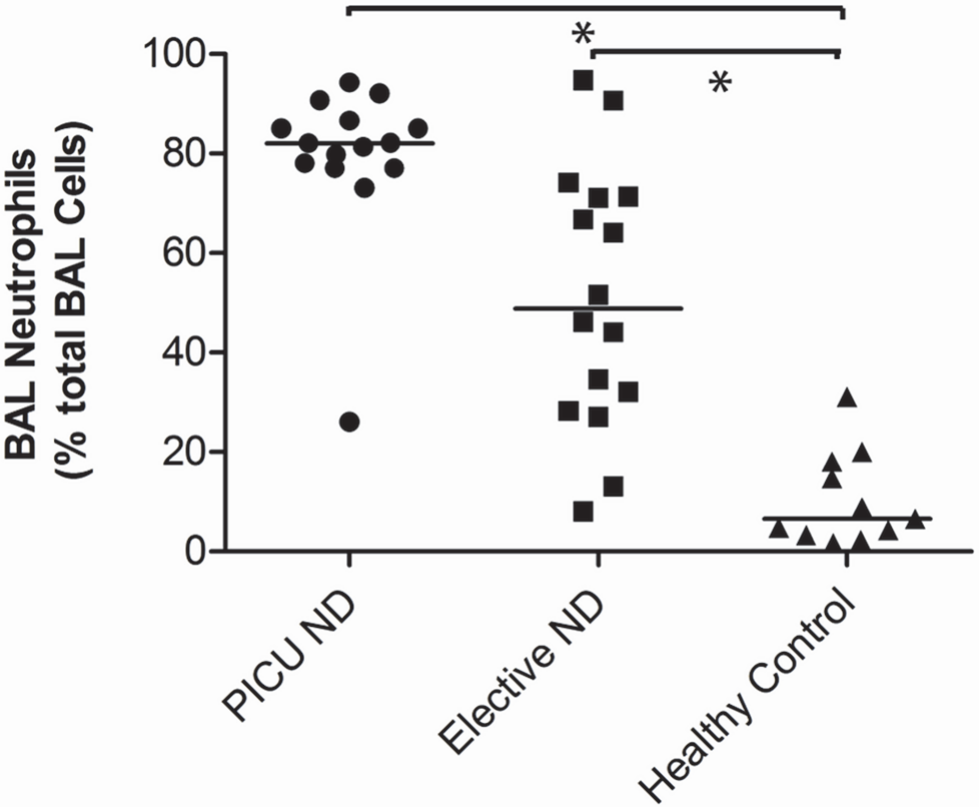
Broncho-alveolar lavage (BAL) neutrophil levels as a percentage of total BAL cells in PICU-ND, Elective-ND and healthy control patients. Elective-ND patients had significant BAL neutrophilia in comparison to healthy controls (p<0.001).

In PICU-ND, Elective-ND and healthy control patients, the median BAL cell yield was 3.9 x 10^6^, 0.9 x 10^6^, and 0.6 x 10^6^ cells/ml respectively (Table 1). Unsurprisingly, a marked neutrophilia was found in PICU-ND BAL (82% [77–86]). However, we also found a higher neutrophil percentage in Elective-ND BALs compared to healthy control samples (Elective-ND, 49% [29–71]; Control, 6.5% [3.3–18]: p<0.001) (Table 1 and Fig 2).

### Cytokine Analysis

Table 1 shows that BAL median [range] IL-8 concentrations were highest in PICU-ND BAL samples (5641[2024–14,703] pg/ml) but were also significantly raised in Elective-ND samples compared to healthy controls (Elective-ND, 646 [0–3633] pg/ml; Control, 0 [0–200] pg/ml: p<0.05).

TGFβ-1 levels were also raised in PICU-ND BAL samples (157 [69–190] pg/ml; Control, 0 [0–0] pg/ml: p<0.001) and Elective-ND patients (Elective-ND, 0 [0–143] pg/ml; p<0.05).

### Correlation of Inflammatory Markers and Respiratory Symptoms

We examined the relationship between total LRSQ-Neuro score and BAL neutrophil percentage, IL-8 and TGF-β1 levels in the Elective-ND group. No statistically significant relationships were found in this small patient cohort (n = 16), although a weak positive trend between total LRSQ-Neuro score and BAL neutrophil percentage was observed (r+0.331, p = 0.221). Notably, those patients with an LRSQ score >20 invariably had an accompanying BAL neutrophilia (data not shown).

### Molzym Sepsitest (16S PCR) Results

We limited our analysis to BAL samples from 18 PICU-ND, 16 Elective-ND and 10 healthy controls patients by 16S PCR because of small sample volumes. We detected microbial DNA in BAL samples from 61% (11/18) PICU-ND patients, 81% (13/16) Elective-ND patients, and 50% (5/10) healthy controls. In PICU-ND patients, microbial DNA was detected in 54% (7/13) of those who had been prescribed antibiotics for >24 hours prior to admission to hospital, and in all patients who had not been prescribed antibiotics.

**Fig 3:**
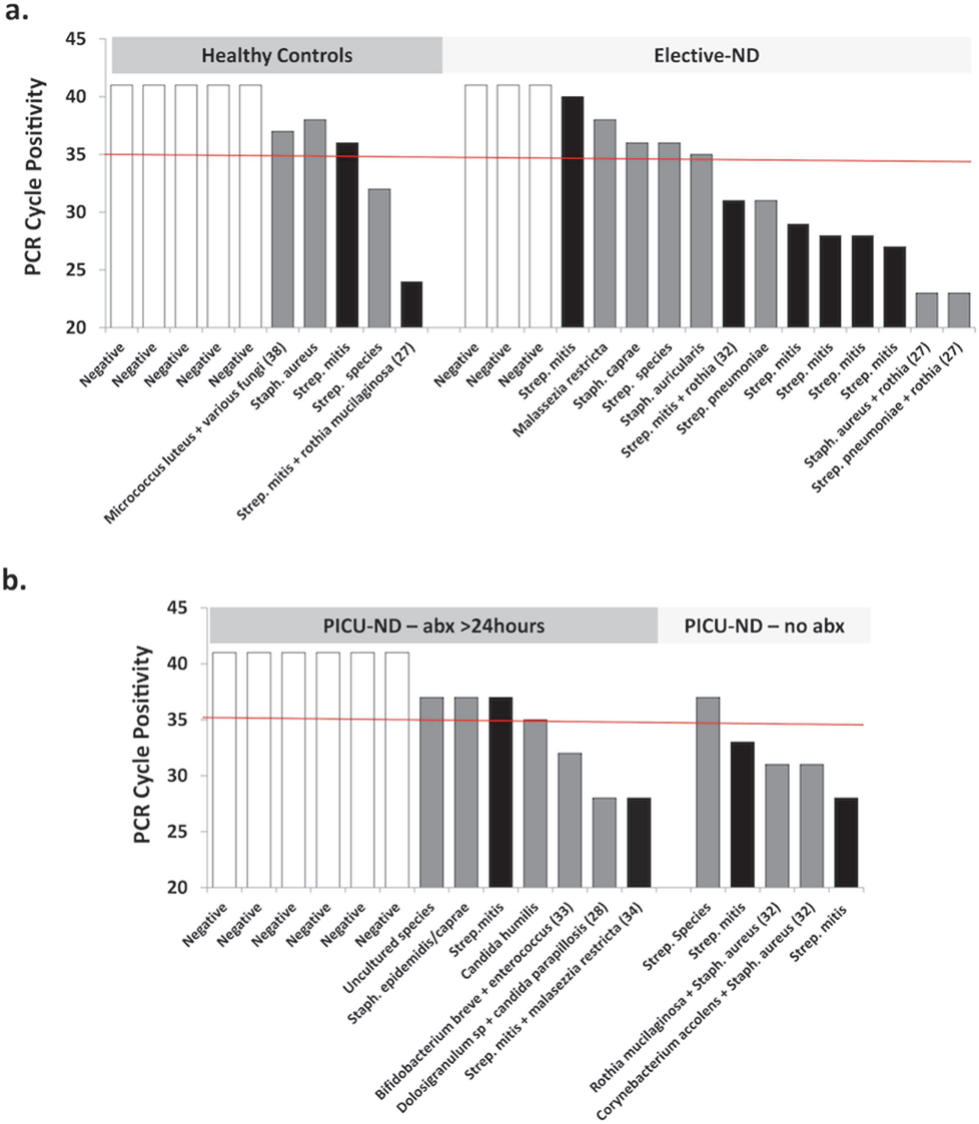
Microbes identified in BAL following Molzym Sepsitest analysis for *a*. Healthy control and Elective-ND patients and *b*. PICU-ND patients who were on antibiotics (abx) for > 24 hours prior to recruitment and PICU-ND patients who had not received antibiotics prior to PICU admission. (White bars, no microbes detected; Black bars, *Streptococcus Mitis*; grey bars, other microbes).

Various, predominantly gram-positive bacteria were identified on sequencing (Fig 3). In both ND groups, microbial DNA from *Staphylococcus aureus*, *Streptococcus mitis* and *Rothia mucilaginosa* were most commonly detected. *Streptococcus mitis* and *Rothia mucilaginosa* were also identified in the two control patients in whom microbial DNA was detected at <cycle <35. For 78% of microbial identifications, sequence coverage was >97%. Sequence coverage was lower in the other 22% due to overlying mixed sequences, resulting in more limited identification at ‘genus’ level (i.e. *Streptococcus* species).

**Fig 4:**
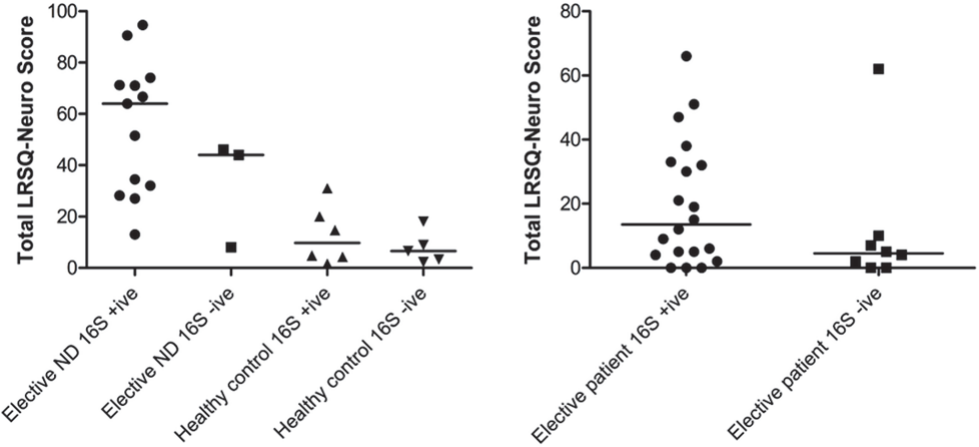
*a*. BAL neutrophil levels and *b*. LRSQ scores in elective BAL samples that were positive and negative for microbial rDNA (m rDNA). Elective BAL samples that were m rDNA positive had significantly higher neutrophil levels than m rDNA negative samples (33%[18–70] vs. 8.5%[4–38] p<0.05). The median total LRSQ-Neuro score was generally higher in the BAL m rDNA positive group (17[5–32] vs. 5[0–9]), but not significantly so (p = 0.097).

Despite *P*. *aeruginosa* being detected on culture of tracheal aspirates from multiple patients prior to this study, it was not detected in BAL samples from any patient using 16S analysis. We also examined the relationship between LRSQ-Neuro score, BAL neutrophilia, and microbial DNA detection, in Elective-ND and control patients (Fig 4). Total LRSQ score was generally higher in patients who had samples that were positive for microbial DNA, although this did not reach statistical significance (+ve, 17[5–32];-ve, 5[0–9]: p = 0.097). Median [range] BAL neutrophil levels were however significantly higher in samples that were microbial DNA positive (+ve, 33 [18–70]%;-ve 8 [4–38]%: p<0.05).

### OprL Data

Given that 12 PICU-ND patients had cultured *P*. *aeruginosa* from their airways previously, and that 5/12 PICU-ND patients had cultured *P*. *aeruginosa* on tracheal aspirate during their current admission to hospital, we sought to confirm our 16S data using an established OprL PCR technique[9,10]. A BAL from a *P*. *aeruginosa* culture-positive cystic fibrosis patient was used as a positive control. *P*. *aeruginosa* was not detected in any PICU-ND, Elective-ND or healthy control BAL sample using this technique (data not shown).

## Discussion

This is the first study to have simultaneously detailed respiratory symptoms, lower airway inflammation and microbiology in individuals with severe ND. We have shown that otherwise well children with ND have significant respiratory symptoms, particularly at night, that affect their families. Furthermore, airway inflammation is found in these same children, and this may relate to levels of chronic respiratory symptomatology. Using 16S rDNA PCR and sequencing analysis technology, we have identified the predominant organisms present within the airways of these children. Of note, *Streptococcus mitis* was found in nearly half of those samples in which an organism was detected, often at high levels. In contrast, *P*. *aeruginosa* was not found in any lower airway sample despite being cultured from other predominantly upper respiratory samples. Lastly, we observed a trend for airway neutrophilia and respiratory symptoms to be greater in those with lower airway microbial DNA positivity, suggesting that lower airway infection may play a significant role in the respiratory morbidity experienced by children with ND.

Although it is well known to paediatric healthcare professionals that children with severe ND often have chronic respiratory symptoms, this is the first time that they have been formally quantified and documented. Whilst it is perhaps no surprise that children admitted to PICU with respiratory deterioration were more symptomatic in the three months leading up to their hospitalisation, it is noteworthy that Elective-ND children, considered well enough by both parents and doctors to undergo general anaesthesia, also had significant respiratory symptoms. *Nighttime* symptoms were especially prominent and predictably correlated with *Effect on Family* scores (p<0.001). Interestingly, *Other* domain scores, which covered extra-thoracic/back of throat symptoms previously shown to be important in predicting aspiration, were also significantly different.[11]

16S rDNA PCR techniques are increasingly being used to characterise the pulmonary microbiome in health and disease.[12] The UMD^-^Liquid test used here differs from other techniques because human and extracellular bacterial DNA is eliminated early in processing, with only those microbes with intact cell walls, sequenced.[8] Our results thus provide a record of the predominant living (growing and non-growing) microorganisms populating the airways of these children at the time of bronchoalveolar lavage. Interestingly, broadly the same microbes, often those generally considered oral commensals, were found in the airways of children with and without severe ND. Where these groups differed was in the timing of PCR cycle positivity. The microbes identified in healthy control samples generally came up after 35 PCR cycles, whereas those within the ND samples came up before 35 cycles. Bacterial load was not formally assessed (using standard curves of bacterial standards at a range of copy number dilutions) and so unfortunately firm conclusions about the relative abundance of microbes between samples cannot be drawn from this study.

The obvious strength of this technique in detecting only intact organisms may also have been a limitation. Prior antibiotic treatment before sampling could have decreased the ability of this test to detect microbial causes of acute symptoms. Of note, no microbes were detected in 6/13 PICU-ND samples from children who had had antibiotics in the preceding 24 hours, whereas they were identified in all 5 PICU patients who did not have antibiotics over this period. Interestingly, we didn’t detect many gram-negative organisms. This may also have been due to the DNA purification step, in which human cells are lysed while bacterial cells ideally remain unaffected. A recognised limitation of these techniques is that bacteria with less robust cell envelopes, such as gram-negative microbes and including *P*. *aeruginosa*, may also be lysed in this step, biasing the reported micro-flora.[13,14]

The microbe identified most frequently was *Streptococcus mitis*. This gram-positive organism is generally considered an oral commensal, and while thought to pose little threat to the immuno-competent, it is a significant cause of endocarditis and septicaemia in neutropenic and immuno-compromised individuals.[15] *Streptococcus mitis* uses a variety of strategies including expression of adhesins, immunoglobulin A proteases and toxins, and modulating the host immune response, to colonise the human oropharynx. In neuro-disabled individuals lacking adequate airway protection, it is possible that these same strategies could also allow the colonisation of the lower respiratory tract.

Given the literature[16,17] and that so many of our cohort of children with ND cultured *P*. *aeruginosa* on cough swab or tracheal aspirate, we were surprised that we did not detect this organism on 16S analysis or OprL PCR. There are a number of explanations for this. It is possible that the sample pre-treatment step in which human and/or microbial extracellular DNA is degraded could have reduced the ability to detect *P*. *aeruginosa* by 16S analysis. However, it should still have been possible to detect it using the conventional PCR assay. It is also possible that *P*. *aeruginosa* was present but at levels below the detection limit of both 16S and PCR assays; it is well recognised that *P*. *aeruginosa* grows easily in the laboratory, and its prior detection may have been due to small numbers of bacteria magnified in culture. It is also well-recognised that microbial growth may differ between lobes of the lung[18] and therefore, it is possible that in ‘blindly’ sampling the lower respiratory tract, we have missed *P*. *aeruginosa* infected lobes. Equally, it is possible that our results are ‘real’ but that previous inferences made on lower respiratory microbiology were actually based on upper airways pseudomonal infection/colonisation; many of these children have regular upper airways suction and poorly coordinated swallows, conditions that provide the perfect opportunity and environment for *P*. *aeruginosa* to be introduced and then to colonise the oropharynx. This potentially important clinical observation should be considered when treating respiratory exacerbations in these children, and warrants further investigation combining these new 16S microbiome profiling techniques with standard culture and PCR to gain a more complete picture of the lower airway microbiome in these patients, and to guide clinicians on the clinical utility of these tests.

It is often difficult to differentiate whether respiratory symptoms originate from the lower or upper respiratory tract in this challenging group of patients. The observation that BAL neutrophil levels were generally higher in those with more respiratory symptoms indicates that at least some of these symptoms originate from the lower respiratory tract. LRSQ-Neuro scores >20 were invariably associated with lower airway neutrophilia, an observation that certainly merits further investigation. It is not inconceivable that this quick, simple score could be used to screen patients who may benefit from specialist respiratory assessment or even as an outcome measure in clinical trials. However, we recognise that for this to happen, further psychometric validation would be required, particularly with regard to sensitivity to change.[19]

The trend for airway neutrophilia and symptoms to be greater in those with lower airway microbial positivity suggests that infection may be the cause of respiratory symptoms and morbidity. While this is difficult to prove with any certainty in our cohort, partly because of the lack of bacterial load data but also because airway neutrophilia can be caused by other conditions such as aspiration, our findings do raise the clinical question as to whether antibiotic prophylaxis would be beneficial in this group of patients. There is a paucity of high quality studies to date and little is known about antibiotic resistance patterns in neuro-disabled children. In our experience, approaches are often inconsistent, with those attending joint neuro-respiratory clinics more likely to be prescribed antibiotic prophylaxis. Formal randomised controlled trials are needed to further assess this, possibly using the LRSQ-Neuro as an outcome measure: development of microbial resistance will be a key consideration in such studies.

## Conclusions

Children with severe ND often have high levels of chronic respiratory symptoms, which may relate to lower airway inflammation. This requires further investigation in a larger patient cohort. Bacterial airway colonisation, particularly with oral commensals, may play a role in both symptom generation and inflammation. Early tailored respiratory assessment of children with ND and chronic respiratory symptoms may be useful and further, well-designed clinical studies are needed to determine the risks and benefits of prophylactic therapy in this group.
